# *N*-[2-(2-Chloro­phen­yl)-2-hy­droxy­eth­yl]propan-2-aminium 4-methyl­benzoate

**DOI:** 10.1107/S1600536810033878

**Published:** 2010-09-25

**Authors:** Hai Feng, Bin Tao Xing, Xin Huang, Ya Jian Zhou, Ying Song

**Affiliations:** a College of Pharmaceutical Sciences, Zhejiang University of Technology, Hangzhou 310014, People’s Republic of China

## Abstract

The title compound, C_11_H_17_ClNO^+^·C_8_H_7_O_2_^−^, was obtained by the reaction of chlorprenaline {or 1-(2-chloro­phen­yl)-2-[(1-methyl­eth­yl)amino]­ethanol} and *p*-toluic acid. The chlorpren­aline is twisted moderately with a C—C—C—C torsion angle of 109.6 (2)°. The two mol­ecules are linked by classical O—H⋯O and N—H⋯O hydrogen bonds. Further N—H⋯O hydrogen bonds link two of these units into dimers.

## Related literature

For related structures, see: Feng *et al.* (2010 ▶); Takwale & Pant (1971 ▶); Tang *et al.* (2009*a* ▶,*b* ▶).
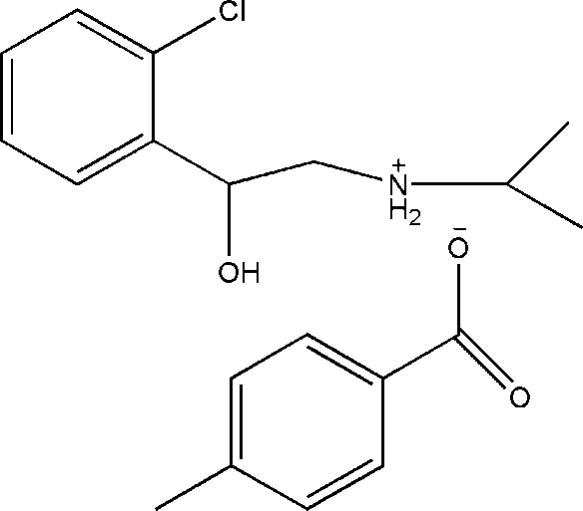


## Experimental

### 

#### Crystal data

C_11_H_17_ClNO^+^·C_8_H_7_O_2_^−^
*M* *_r_* = 349.84
Monoclinic, 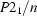

*a* = 8.5966 (4) Å
*b* = 8.1288 (3) Å
*c* = 26.8949 (12) Å
β = 91.600 (1)°
*V* = 1878.68 (14) Å^3^
*Z* = 4
Mo *K*α radiation
μ = 0.22 mm^−1^
*T* = 296 K
0.37 × 0.30 × 0.22 mm

#### Data collection

Rigaku R-AXIS RAPID/ZJUG CCD diffractometer
Absorption correction: multi-scan (*ABSCOR*; Higashi, 1995 ▶) *T*_min_ = 0.913, *T*_max_ = 0.953
27924 measured reflections
4271 independent reflections
2763 reflections with *I* > 2σ(*I*)
*R*_int_ = 0.036

#### Refinement

*R*[*F* ^2^ > 2σ(*F* ^2^)] = 0.042
*wR*(*F* ^2^) = 0.125
*S* = 1.00
4271 reflections
222 parameters
H-atom parameters constrained
Δρ_max_ = 0.21 e Å^−3^
Δρ_min_ = −0.31 e Å^−3^

### 

Data collection: *PROCESS-AUTO* (Rigaku/MSC, 2006 ▶); cell refinement: *PROCESS-AUTO*; data reduction: *CrystalStructure* (Rigaku/MSC, 2007 ▶); program(s) used to solve structure: *SHELXS97* (Sheldrick, 2008 ▶); program(s) used to refine structure: *SHELXL97* (Sheldrick, 2008 ▶); molecular graphics: *ORTEP-3* (Farrugia, 1997 ▶); software used to prepare material for publication: *WinGX* (Farrugia, 1999 ▶).

## Supplementary Material

Crystal structure: contains datablocks global, I. DOI: 10.1107/S1600536810033878/rk2225sup1.cif

Structure factors: contains datablocks I. DOI: 10.1107/S1600536810033878/rk2225Isup2.hkl

Additional supplementary materials:  crystallographic information; 3D view; checkCIF report

## Figures and Tables

**Table 1:** Hydrogen-bond geometry (Å, °)

*D*—H⋯*A*	*D*—H	H⋯*A*	*D*⋯*A*	*D*—H⋯*A*
O1—H101⋯O3	0.82	1.88	2.6986 (18)	173
N1—H103⋯O2	0.90	1.91	2.7835 (18)	164
N1—H102⋯O3^i^	0.90	1.89	2.7824 (19)	174

Symmetry code: (i) 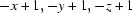.

**Fig. 1.**
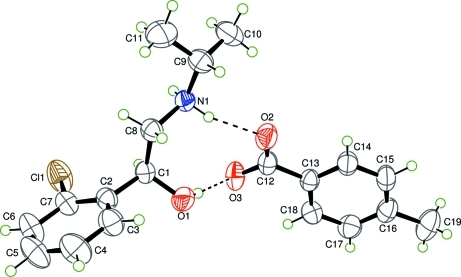
The asymmetric unit of I with atom numbering scheme. Displacement ellipsoids are presented at 50% probability level. H atoms are presented as a small spheres of arbitrary radius.

**Fig. 2.**
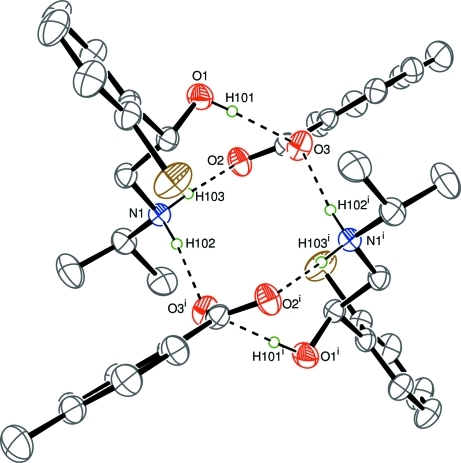
The two units of I with atom labels. The dashed lines indicate hydrogen bonds.

## supplementary crystallographic information

### Comment

A recent study reports the structure of bis{*N*-[2-(2-chlorophenyl)-2-hydroxyethyl]propan-2-aminium} oxalate (Tang *et al.*, 2009*b*), which was synthesized by oxalic acid and chlorprenaline (Tang *et al.*, 2009*a*). Here using *p*-toluic acid instead of oxalic acid and following a similar synthetic procedure yields the title compound, I.

In I, the chlorprenaline molecule and the *p*-toluic molecule are linked to each other by the classical N1—H103···O2 hydogen bond [2.7835 (18)Å] and the O1—H101···O3 hydogen bond [2.6986 (18)Å] (Fig. 1 & Table 1). The chlorprenaline in I are twisted moderately as compared with those of other compounds. The C7—C2—C1—C8 torsion angle of 109.6 (2)° is larger than the value of the similar torsion angle of 91.9 (2)° (Tang *et al.*, 2009*a*). The C12–O2 distance of 1.248 (2)Å is much shorter than the similar distance of 1.292 (8)Å (Takwale & Pant, 1971). The C9–N1 distance of 1.507 (2)Å is longer than the value of the similar bond distance of 1.473 (4)Å (Tang *et al.*, 2009*b*), as similar as the value of the similar bond distance of 1.503 (2)Å (Feng *et al.*, 2010).

Classical O—H···O and N—H···O hydrogen bonds are found in the cystal structure (Fig. 2) are essential forces in crystal formation.

### Experimental

Racemic chlorprenaline was prepared by chlorprenaline hydrochloride purchased from ShangHai Shengxin Medicine & Chemical Co., Ltd. ShangHai, China. Chlorprenaline hydrochloride and NaOH in a molar ratio of 1:1 were mixed and dissolved in a methanol–water solution (1:1 *v*/*v*). The precipitate formed was filtered off, washed with water and dried. It was used without further purification. Racemic chlorprenaline (0.5 g, 0.0023 mol) was dissolved in methanol (7 ml) and then *p*-toluylic acid (0.31 g, 0.0023 mol) was added.The mixture was dissovled by heating to 343 K where a clear solution resulted. The resulting solution was concentrated at ambient temperature. Colourless crystals of title compound separated from the solution in about 70% yield after one day.

### Refinement

All of the H atoms were placed in calculated positions and allowed to ride on their parent atoms at distances of 0.93Å (aromatic), 0.98Å (methine), 0.97Å (methylene), 0.96Å (methyl) 0.82Å (hydroxyl) and 0.90Å (amine), with *U*_iso_(H) = 1.2–1.5 *U*_eq_(parent atom).

### Crystal data

**Table:**

C_11_H_17_ClNO^+^·C_8_H_7_O_2_^−^	*F*(000) = 744
*M**_r_* = 349.84	*D*_x_ = 1.237 Mg m^−^^3^
Monoclinic, *P*2_1_/*n*	Mo *K*α radiation, λ = 0.71073 Å
Hall symbol: -P 2yn	Cell parameters from 17098 reflections
*a* = 8.5966 (4) Å	θ = 3.0–27.4°
*b* = 8.1288 (3) Å	µ = 0.22 mm^−^^1^
*c* = 26.8949 (12) Å	*T* = 296 K
β = 91.600 (1)°	Chunk, colourless
*V* = 1878.68 (14) Å^3^	0.37 × 0.30 × 0.22 mm
*Z* = 4	

### Data collection

**Table:**

Rigaku R-AXIS RAPID/ZJUG CCD diffractometer	4271 independent reflections
Radiation source: rotate anode	2763 reflections with *I* > 2σ(*I*)
graphite	*R*_int_ = 0.036
Detector resolution: 10.00 pixels mm^-1^	θ_max_ = 27.4°, θ_min_ = 3.0°
φ and ω scans	*h* = −11→11
Absorption correction: multi-scan (*ABSCOR*; Higashi, 1995)	*k* = −10→9
*T*_min_ = 0.913, *T*_max_ = 0.953	*l* = −34→34
27924 measured reflections	

### Refinement

**Table:**

Refinement on *F*^2^	Secondary atom site location: difference Fourier map
Least-squares matrix: full	Hydrogen site location: inferred from neighbouring sites
*R*[*F*^2^ > 2σ(*F*^2^)] = 0.042	H-atom parameters constrained
*wR*(*F*^2^) = 0.125	*w* = 1/[σ^2^(*F*_o_^2^) + (0.0502*P*)^2^ + 0.6753*P*] where *P* = (*F*_o_^2^ + 2*F*_c_^2^)/3
*S* = 1.00	(Δ/σ)_max_ < 0.001
4271 reflections	Δρ_max_ = 0.21 e Å^−^^3^
222 parameters	Δρ_min_ = −0.31 e Å^−^^3^
0 restraints	Extinction correction: *SHELXL97* (Sheldrick, 2008), Fc^*^=kFc[1+0.001xFc^2^λ^3^/sin(2θ)]^-1/4^
Primary atom site location: structure-invariant direct methods	Extinction coefficient: 0.0084 (10)

### Special details

**Table:**

Geometry. All s.u.'s (except the s.u. in the dihedral angle between two l.s. planes) are estimated using the full covariance matrix. The cell s.u.'s are taken into account individually in the estimation of s.u.'s in distances, angles and torsion angles; correlations between s.u.'s in cell parameters are only used when they are defined by crystal symmetry. An approximate (isotropic) treatment of cell s.u.'s is used for estimating s.u.'s involving l.s. planes.
Refinement. Refinement of *F*^2^ against ALL reflections. The weighted *R*-factor *wR* and goodness of fit *S* are based on *F*^2^, conventional *R*-factors *R* are based on *F*, with *F* set to zero for negative *F*^2^. The threshold expression of *F*^2^ > σ(*F*^2^) is used only for calculating *R*-factors(gt) *etc*. and is not relevant to the choice of reflections for refinement. *R*-factors based on *F*^2^ are statistically about twice as large as those based on *F*, and *R*-factors based on ALL data will be even larger.

### Fractional atomic coordinates and isotropic or equivalent isotropic displacement parameters (Å^2^)

**Table:**

	*x*	*y*	*z*	*U*_iso_*/*U*_eq_	
Cl1	0.29951 (9)	0.54161 (7)	0.65218 (2)	0.0833 (2)	
O1	0.27619 (16)	0.21200 (19)	0.53119 (5)	0.0615 (4)	
H101	0.2803	0.2754	0.5076	0.092*	
O2	0.49211 (17)	0.21720 (18)	0.43539 (5)	0.0619 (4)	
N1	0.62036 (16)	0.27020 (17)	0.53021 (5)	0.0420 (3)	
H102	0.6383	0.3783	0.5348	0.050*	
H103	0.5619	0.2592	0.5021	0.050*	
C7	0.2495 (2)	0.3348 (2)	0.65680 (7)	0.0541 (5)	
C1	0.3646 (2)	0.2763 (2)	0.57188 (6)	0.0440 (4)	
H1	0.3678	0.3966	0.5696	0.053*	
C2	0.2854 (2)	0.2261 (2)	0.61907 (6)	0.0459 (4)	
C8	0.5294 (2)	0.2082 (2)	0.57268 (6)	0.0486 (4)	
H8A	0.5820	0.2392	0.6037	0.058*	
H8B	0.5252	0.0891	0.5713	0.058*	
C9	0.7739 (2)	0.1855 (3)	0.52294 (8)	0.0571 (5)	
H9	0.7533	0.0726	0.5118	0.068*	
C4	0.1703 (3)	0.0101 (3)	0.66772 (10)	0.0788 (7)	
H4	0.1439	−0.1001	0.6714	0.095*	
C6	0.1758 (3)	0.2841 (3)	0.69932 (8)	0.0749 (7)	
H6	0.1540	0.3592	0.7243	0.090*	
C10	0.8567 (3)	0.2759 (3)	0.48204 (9)	0.0744 (6)	
H10A	0.7901	0.2810	0.4528	0.112*	
H10B	0.9507	0.2186	0.4745	0.112*	
H10C	0.8817	0.3855	0.4930	0.112*	
C3	0.2444 (2)	0.0620 (3)	0.62573 (8)	0.0607 (5)	
H3	0.2677	−0.0143	0.6013	0.073*	
C11	0.8699 (3)	0.1785 (4)	0.57050 (10)	0.0913 (8)	
H11A	0.8804	0.2873	0.5841	0.137*	
H11B	0.9711	0.1353	0.5638	0.137*	
H11C	0.8196	0.1086	0.5939	0.137*	
C5	0.1353 (3)	0.1218 (3)	0.70422 (9)	0.0830 (8)	
H5	0.0840	0.0871	0.7323	0.100*	
O3	0.30804 (17)	0.40215 (16)	0.44986 (5)	0.0589 (4)	
C13	0.3104 (2)	0.2771 (2)	0.37008 (6)	0.0439 (4)	
C12	0.3761 (2)	0.3002 (2)	0.42202 (6)	0.0467 (4)	
C14	0.3905 (2)	0.1855 (2)	0.33582 (7)	0.0547 (5)	
H14	0.4842	0.1360	0.3453	0.066*	
C18	0.1687 (2)	0.3448 (2)	0.35524 (7)	0.0529 (5)	
H18	0.1117	0.4052	0.3778	0.064*	
C16	0.1925 (2)	0.2349 (3)	0.27234 (7)	0.0578 (5)	
C15	0.3330 (3)	0.1666 (3)	0.28760 (7)	0.0609 (5)	
H15	0.3901	0.1067	0.2650	0.073*	
C17	0.1113 (2)	0.3231 (3)	0.30701 (7)	0.0607 (5)	
H17	0.0157	0.3690	0.2978	0.073*	
C19	0.1321 (4)	0.2139 (4)	0.21933 (9)	0.0890 (8)	
H19A	0.2018	0.2667	0.1971	0.133*	
H19B	0.0308	0.2629	0.2158	0.133*	
H19C	0.1254	0.0989	0.2115	0.133*	

### Atomic displacement parameters (Å^2^)

**Table:**

	*U*^11^	*U*^22^	*U*^33^	*U*^12^	*U*^13^	*U*^23^
Cl1	0.1200 (5)	0.0570 (3)	0.0746 (4)	−0.0060 (3)	0.0327 (3)	−0.0103 (3)
O1	0.0601 (8)	0.0804 (10)	0.0435 (7)	−0.0112 (7)	−0.0052 (6)	0.0058 (7)
O2	0.0651 (9)	0.0754 (9)	0.0446 (7)	0.0106 (7)	−0.0091 (6)	−0.0056 (7)
N1	0.0427 (8)	0.0434 (8)	0.0400 (7)	0.0015 (6)	0.0020 (6)	−0.0029 (6)
C7	0.0561 (11)	0.0568 (11)	0.0498 (10)	0.0044 (9)	0.0102 (8)	0.0035 (9)
C1	0.0488 (10)	0.0454 (9)	0.0378 (8)	0.0003 (7)	0.0026 (7)	0.0009 (7)
C2	0.0460 (10)	0.0515 (10)	0.0404 (9)	0.0035 (8)	0.0026 (7)	0.0067 (8)
C8	0.0497 (10)	0.0523 (10)	0.0440 (9)	0.0024 (8)	0.0034 (8)	0.0064 (8)
C9	0.0487 (11)	0.0585 (12)	0.0643 (12)	0.0116 (9)	0.0063 (9)	−0.0040 (9)
C4	0.0927 (17)	0.0631 (14)	0.0818 (16)	−0.0038 (12)	0.0234 (13)	0.0242 (12)
C6	0.0881 (17)	0.0820 (16)	0.0561 (12)	0.0105 (13)	0.0285 (12)	0.0043 (11)
C10	0.0552 (13)	0.0929 (17)	0.0761 (15)	0.0067 (12)	0.0194 (11)	−0.0026 (13)
C3	0.0708 (13)	0.0549 (12)	0.0570 (12)	−0.0008 (10)	0.0103 (10)	0.0072 (9)
C11	0.0580 (14)	0.131 (2)	0.0845 (17)	0.0235 (15)	−0.0098 (12)	0.0098 (16)
C5	0.0928 (18)	0.0889 (18)	0.0691 (15)	0.0057 (14)	0.0357 (13)	0.0274 (13)
O3	0.0889 (10)	0.0460 (7)	0.0420 (7)	0.0038 (7)	0.0029 (6)	−0.0067 (6)
C13	0.0538 (10)	0.0406 (9)	0.0375 (8)	−0.0040 (7)	0.0015 (7)	0.0012 (7)
C12	0.0616 (11)	0.0399 (9)	0.0388 (9)	−0.0050 (8)	0.0023 (8)	−0.0008 (7)
C14	0.0567 (11)	0.0613 (12)	0.0460 (10)	0.0066 (9)	−0.0016 (8)	−0.0078 (9)
C18	0.0578 (12)	0.0537 (11)	0.0475 (10)	0.0037 (9)	0.0053 (8)	0.0015 (8)
C16	0.0710 (13)	0.0594 (12)	0.0426 (10)	−0.0094 (10)	−0.0061 (9)	0.0016 (9)
C15	0.0716 (14)	0.0698 (13)	0.0414 (10)	0.0016 (11)	0.0039 (9)	−0.0116 (9)
C17	0.0597 (12)	0.0664 (13)	0.0555 (11)	0.0027 (10)	−0.0051 (9)	0.0076 (10)
C19	0.114 (2)	0.0984 (19)	0.0531 (13)	−0.0079 (16)	−0.0230 (13)	−0.0049 (13)

### Geometric parameters (Å, °)

**Table:**

Cl1—C7	1.741 (2)	C10—H10A	0.9600
O1—C1	1.415 (2)	C10—H10B	0.9600
O1—H101	0.8200	C10—H10C	0.9600
O2—C12	1.248 (2)	C3—H3	0.9300
N1—C8	1.490 (2)	C11—H11A	0.9600
N1—C9	1.507 (2)	C11—H11B	0.9600
N1—H102	0.9000	C11—H11C	0.9600
N1—H103	0.9000	C5—H5	0.9300
C7—C6	1.386 (3)	O3—C12	1.271 (2)
C7—C2	1.387 (3)	C13—C14	1.383 (2)
C1—C2	1.513 (2)	C13—C18	1.386 (3)
C1—C8	1.521 (2)	C13—C12	1.504 (2)
C1—H1	0.9800	C14—C15	1.384 (3)
C2—C3	1.392 (3)	C14—H14	0.9300
C8—H8A	0.9700	C18—C17	1.386 (3)
C8—H8B	0.9700	C18—H18	0.9300
C9—C11	1.504 (3)	C16—C17	1.381 (3)
C9—C10	1.517 (3)	C16—C15	1.382 (3)
C9—H9	0.9800	C16—C19	1.513 (3)
C4—C5	1.376 (4)	C15—H15	0.9300
C4—C3	1.379 (3)	C17—H17	0.9300
C4—H4	0.9300	C19—H19A	0.9600
C6—C5	1.371 (4)	C19—H19B	0.9600
C6—H6	0.9300	C19—H19C	0.9600
			
C1—O1—H101	109.5	H10A—C10—H10C	109.5
C8—N1—C9	115.19 (14)	H10B—C10—H10C	109.5
C8—N1—H102	108.5	C4—C3—C2	121.5 (2)
C9—N1—H102	108.5	C4—C3—H3	119.2
C8—N1—H103	108.5	C2—C3—H3	119.2
C9—N1—H103	108.5	C9—C11—H11A	109.5
H102—N1—H103	107.5	C9—C11—H11B	109.5
C6—C7—C2	122.1 (2)	H11A—C11—H11B	109.5
C6—C7—Cl1	117.75 (17)	C9—C11—H11C	109.5
C2—C7—Cl1	120.19 (14)	H11A—C11—H11C	109.5
O1—C1—C2	107.74 (14)	H11B—C11—H11C	109.5
O1—C1—C8	110.88 (15)	C6—C5—C4	120.3 (2)
C2—C1—C8	109.32 (14)	C6—C5—H5	119.8
O1—C1—H1	109.6	C4—C5—H5	119.8
C2—C1—H1	109.6	C14—C13—C18	118.17 (16)
C8—C1—H1	109.6	C14—C13—C12	120.33 (16)
C7—C2—C3	117.01 (17)	C18—C13—C12	121.50 (16)
C7—C2—C1	123.81 (17)	O2—C12—O3	124.06 (16)
C3—C2—C1	119.18 (17)	O2—C12—C13	118.45 (16)
N1—C8—C1	112.01 (14)	O3—C12—C13	117.49 (16)
N1—C8—H8A	109.2	C13—C14—C15	120.84 (18)
C1—C8—H8A	109.2	C13—C14—H14	119.6
N1—C8—H8B	109.2	C15—C14—H14	119.6
C1—C8—H8B	109.2	C13—C18—C17	120.41 (18)
H8A—C8—H8B	107.9	C13—C18—H18	119.8
C11—C9—N1	111.64 (17)	C17—C18—H18	119.8
C11—C9—C10	112.2 (2)	C17—C16—C15	117.54 (17)
N1—C9—C10	107.65 (16)	C17—C16—C19	121.9 (2)
C11—C9—H9	108.4	C15—C16—C19	120.6 (2)
N1—C9—H9	108.4	C16—C15—C14	121.36 (19)
C10—C9—H9	108.4	C16—C15—H15	119.3
C5—C4—C3	119.8 (2)	C14—C15—H15	119.3
C5—C4—H4	120.1	C16—C17—C18	121.64 (19)
C3—C4—H4	120.1	C16—C17—H17	119.2
C5—C6—C7	119.2 (2)	C18—C17—H17	119.2
C5—C6—H6	120.4	C16—C19—H19A	109.5
C7—C6—H6	120.4	C16—C19—H19B	109.5
C9—C10—H10A	109.5	H19A—C19—H19B	109.5
C9—C10—H10B	109.5	C16—C19—H19C	109.5
H10A—C10—H10B	109.5	H19A—C19—H19C	109.5
C9—C10—H10C	109.5	H19B—C19—H19C	109.5
			
C6—C7—C2—C3	−0.2 (3)	C1—C2—C3—C4	−179.13 (19)
Cl1—C7—C2—C3	178.83 (15)	C7—C6—C5—C4	1.2 (4)
C6—C7—C2—C1	179.50 (19)	C3—C4—C5—C6	−0.9 (4)
Cl1—C7—C2—C1	−1.4 (3)	C14—C13—C12—O2	−9.8 (3)
O1—C1—C2—C7	−129.79 (18)	C18—C13—C12—O2	169.70 (18)
C8—C1—C2—C7	109.6 (2)	C14—C13—C12—O3	171.21 (17)
O1—C1—C2—C3	49.9 (2)	C18—C13—C12—O3	−9.3 (3)
C8—C1—C2—C3	−70.6 (2)	C18—C13—C14—C15	2.1 (3)
C9—N1—C8—C1	−169.34 (15)	C12—C13—C14—C15	−178.45 (18)
O1—C1—C8—N1	68.35 (19)	C14—C13—C18—C17	−1.1 (3)
C2—C1—C8—N1	−173.02 (14)	C12—C13—C18—C17	179.36 (17)
C8—N1—C9—C11	−50.6 (2)	C17—C16—C15—C14	0.0 (3)
C8—N1—C9—C10	−174.10 (16)	C19—C16—C15—C14	179.6 (2)
C2—C7—C6—C5	−0.7 (4)	C13—C14—C15—C16	−1.5 (3)
Cl1—C7—C6—C5	−179.8 (2)	C15—C16—C17—C18	0.9 (3)
C5—C4—C3—C2	−0.1 (4)	C19—C16—C17—C18	−178.6 (2)
C7—C2—C3—C4	0.6 (3)	C13—C18—C17—C16	−0.3 (3)

### Hydrogen-bond geometry (Å, °)

**Table:**

*D*—H···*A*	*D*—H	H···*A*	*D*···*A*	*D*—H···*A*
O1—H101···O3	0.82	1.88	2.6986 (18)	173
N1—H103···O2	0.90	1.91	2.7835 (18)	164
N1—H102···O3^i^	0.90	1.89	2.7824 (19)	174

Symmetry codes: (i) −*x*+1, −*y*+1, −*z*+1.
